# Brain Aging and Electrophysiological Signaling: Revisiting the Spreading Depression Model

**DOI:** 10.3389/fnagi.2019.00136

**Published:** 2019-06-07

**Authors:** Rubem Carlos Araújo Guedes, Ricardo Abadie-Guedes

**Affiliations:** ^1^Departamento de Nutrição, Universidade Federal de Pernambuco, Recife, Brazil; ^2^Departamento de Fisiologia e Farmacologia, Universidade Federal de Pernambuco, Recife, Brazil

**Keywords:** aging, alcohol abuse, brain excitability, exercise, malnutrition, spreading depression

## Abstract

As a consequence of worldwide improvement in health care, the aging portion of the human population has increased, now representing a higher proportion of the total population. This fact raises great concern regarding how to age while maintaining good brain function. Very often, alterations in brain electrophysiological signaling are associated with age-dependent functional disorders of the brain. Therefore, animal models suitable for the study of age-related changes in electrical activity of the brain can be very useful. Herein, we review changes in brain electrophysiological features as a function of age by analyzing studies in the rat brain on the phenomenon known as cortical spreading depression (CSD). Alterations in the brain’s capability to generate and propagate CSD may be related to differences in the propensity to develop certain neurological diseases, such as epilepsy, stroke, and migraine, which can biunivocally interact with the aging process. In this review, we revisit ours and others’ previous studies on electrophysiological features of the CSD phenomenon, such as its velocity of propagation and amplitude and duration of its slow negative DC shift, as a function of the animal age, as well as the interaction between age and other factors, such as ethanol consumption, physical exercise, and nutritional status. In addition, we discuss one relatively new feature through which CSD modulates brain signaling: the ability to potentiate the brain’s spontaneous electrical activity. We conclude that the CSD model might importantly contribute to a better understanding of the aging/brain signaling relationship.

## Introduction: Aging and Brain Function

A growing percentage of the human population is presently living longer than in past centuries. Because of this, the current health authorities are increasingly concerned about how to maintain normal functionality while becoming older (World Health Organization [WHO], 2015), and this includes avoiding, or at least minimizing, the negative effects of the aging process on brain function. Compared with younger people, the elderly population is at a higher risk for stroke (Zhou et al., 2018), disturbances of neurovascular processes (Bell et al., 2010), single cell structure and electric alterations (Coskren et al., 2015), and cognitive function disorders (Hernandez et al., 2018). Evidence also suggests that brain-guided sensory-motor activity can be substantially impaired by the aging process (Anguera and Gazzaley, 2012; Bernard and Seidler, 2014).

In this review, we revisit the theme of the influence of aging on electrophysiological aspects of brain signaling as indexed by the excitability-related phenomenon known as cortical spreading depression (CSD; Guedes et al., 1996; Farkas et al., 2011; Sousa et al., 2018). In addition, we examine how aging interacts with other factors, such as alcohol consumption (Abadie-Guedes et al., 2016), physical exercise, and nutritional status (Batista-de-Oliveira et al., 2012; Silva-Gondim et al., 2017), regarding the brain’s capability of generating and propagating CSD, which has been continuously investigated in our laboratory for many years (see Guedes, 2005, 2011; Abadie-Guedes et al., 2016, for an overview). Using the CSD phenomenon, we previously studied the action of chronic ethanol administration via gavage on treadmill/swimming exercise and early nutritional deficiencies in the brain of albino rats at various ages. Our findings support and highlight the usefulness of the CSD model in studying age-related alterations in the brain’s electrophysiological features, as we will detail in the subsequent sections of this review.

## Influence of Aging on the Brain’s Electrical Activity

In mammals, including humans, cells in the normal brain exert their various functions by generating and propagating electrical activity. Digitalization, storage, and analysis of brain electrical signals can be achieved by electronic devices based on microchip-operated circuits and appropriate software programs (Guedes, 2005; Coskren et al., 2015; Silva-Gondim et al., 2017). In many clinical cases, detection of an altered EEG-pattern helps diagnose epileptic and non-epileptic neurological disorders (Asadi-Pooya et al., 2017). Studies on the features of brain electrical activity have indicated that some alterations are age-dependent. For example, in Rhesus monkeys, somatosensory, visual, and auditory evoked electric responses are delayed in aged animals compared to young controls (Ibáñez-Contreras et al., 2018), and computational models using data from whole cell patch-clamp recordings and high-resolution digital reconstruction of neurons suggest increased firing rates in aged subjects (Coskren et al., 2015). Studies in humans also demonstrate age-related EEG (Watson et al., 2012) and evoked potential changes (Bourisly and Shuaib, 2018). Supplementary Table S1 presents examples of brain morphological and physiological features that change as a function of aging.

The effects of aging on the brain’s electrical activity are attributed to several factors, including alterations in brain vasculature (Guo et al., 2018; McKetton et al., 2018) and transmitter systems (Schliebs and Arendt, 2011; Hernandez et al., 2018), as well as free radical-induced injury to brain cells as a consequence of redox imbalance (Carvalho and Moreira, 2018), among others. The impact of redox imbalance is considerably significant, as the brain demands as much as 20% of an organism’s oxygen in order to function normally (Guillemin et al., 2017). This implies a high susceptibility of brain cells to oxidative injury (Guillemin et al., 2017), which seems to increase the risk for cerebrovascular-based brain diseases (Seals et al., 2014).

## Electrophysiological Effects of Aging: Studies Using the Spreading Depression Model

The phenomenon known as cortical spreading depression (CSD) was first described by the Brazilian scientist Aristides A. P. Leão (1944a). During the recording of brain electrical activity in anesthetized rabbits, Leão first noted that a strong electric stimulation of a point on the cortical surface produced a reversible reduction (depression) of the electrocorticographic waves, which recovers completely after a few minutes. From the stimulated cortical point, CSD reversibly propagates in all directions to increasingly remote cortical regions. In a second publication in the same year (Leão, 1944b), this author documented that vasodilation of cortical blood vessels accompanied CSD. Three years later (Leão, 1947), the author described a direct current (DC) slow potential change that appeared in the depressed cortical tissue and that was measured in relation to a remote reference point. This slow, all-or-none type DC signal is considered the hallmark of CSD. According to current data on animal and human experiments, the brain’s electrical silence of CSD is caused by neuronal and glial depolarization (Hartings et al., 2017). CSD propagation velocity over the cortical tissue is paradoxically very low, on the order of 2–5 mm/min, in contrast with the much faster propagation velocity of neuronal action potentials, on the order of tens of m/s (Guedes et al., 2016). SD has been documented in all species so far investigated, from fishes (Young, 1980) to humans (Lauritzen and Strong, 2017), which suggests a role for this phenomenon in the normal brain. On the other hand, CSD has been related to brain pathophysiology (Lauritzen and Strong, 2017; Dreier et al., 2018). Some important neurological disorders are postulated as having CSD causally participating in their pathogenesis. This is the case for migraines, traumatic brain injury (Lauritzen, 1987; Hartings et al., 2009, 2011; Torrente et al., 2014; Sadeghian et al., 2018; Vinogradova, 2018), subarachnoid hemorrhage (Dreier et al., 2006, 2009) epilepsy (Guedes and Cavalheiro, 1997; Mesgari et al., 2017), and stroke (Takano et al., 1996; see Lauritzen and Strong, 2017 for an overview). A number of nutritional, pharmacologic, environmental, and hormonal manipulations are shown to either increase or decrease CSD velocity of propagation compared to control animals (Guedes, 2011; Guedes et al., 2017). These pieces of evidence support the conclusion that CSD velocity of propagation is a useful index to evaluate electrophysiological aspects of brain signaling. Table 1 presents some animal and human studies on CSD characteristics as a function of age.

**Table 1:** Some studies involving aging and CSD.

Examples of studies illustrating some effects of aging on spreading depression			

Species	References (in alphabetical order)	Condition	Main outcomes
Rat	Abadie-Guedes et al., 2016	The authors recorded CSD in rats of two age-ranges that received acute or chronic ethanol (E), with or without alpha-tocopherol (T)	Increasing age decelerated CSD. E influenced CSD on both ages. T counteracts the ethanol effects on CSD
Rat	Barreto et al., 2014	The authors reviewed the action of ethanol consumption on CSD	The authors postulate that some of the ethanol effects are related with mitochondrial dysfunction with aging
Rat	Batista-de-Oliveira et al., 2012	The authors investigated whether physical exercise, lactation conditions, and aging interact and modulate brain electrophysiology as indexed by CSD	An aging-related CSD deceleration was observed. Unfavorable lactation facilitated CSD, while treadmill exercise impaired it. Failure to elicit CSD by KCl was greater in older rats
Rat	Clark et al., 2014	The authors characterized the SD in young, middle-aged, and old rats after middle cerebral artery occlusion, utilizing multimodal imaging with voltage-sensitive dyes	Age reduced the number of SDs but increased the size of ischemic area displaying prolonged SD. The growth of area generating SD positively correlated with the ischemic core area
Human	Fabricius et al., 2006	Electrographic recordings that were identical to Leao’s CSD, were recorded in acutely brain-injured human patients with various ages	CSDs in acute brain disorders occur at lower incidence in older patients than in younger ones
Various	Farkas and Bari, 2014	The author reviewed studies on animals and humans relating the SD in the ischemic brain with the age of the organism	The authors hypothesize that augmentation of the ischemic lesion in the elderly patients is age-dependent
Rat	Farkas et al., 2011	CSD recording in rats of various ages and brain hypoperfusion	The data suggested a reduced sensitivity of the cortex to CSD elicitation with early aging, and a less responsive cerebrovascular system with chronic hypoperfusion
Rat	Guedes et al., 1996	Evaluation of cortical spreading depression (CSD) propagation in rats and gerbils of various ages	In both species, older animals displayed lower CSD velocities than younger ones; deficiency of dietary antioxidant vitamins abolished this effect
Rat	Hertelendy et al., 2017	In 38 rats (7–30 week-old), CSD was documented by ECoG and DC potential recording	Advancing age and ischemia elevate the electric threshold to elicit CSD
Rat	Menyhárt et al., 2015	KCl-induced SD after bilateral common carotid artery occlusion was studied in young and old rats. ECoG, DC potential, and cerebral blood flow (CBF) variations were acquired	Ischemia and age delayed the recovery from SD. CBF decreased during ischemia in the old animals, and inverse neurovascular coupling with SD evolved in the old ischemic group
Rat	Menyhárt et al., 2017	CSD was recorded in 2-months, and 18–20 months old rats under ischemia and reperfusion	Old age changes CSD parameters and its relation with ischemia, suggesting a kind of metabolic dysfunction in the older brain
Rat	Sousa et al., 2018	Adult young and aged rats received a powder extract of murici fruit, which is antioxidant. CSD was recorded and oxidative stress was biochemically evaluated	Aging decreased CSD propagation, catalase activity and glutathione/oxidized glutathione ratio; increased malondialdehyde concentrations and SOD activity


### Age Correlates Negatively With CSD Propagation

**FIGURE 1:**
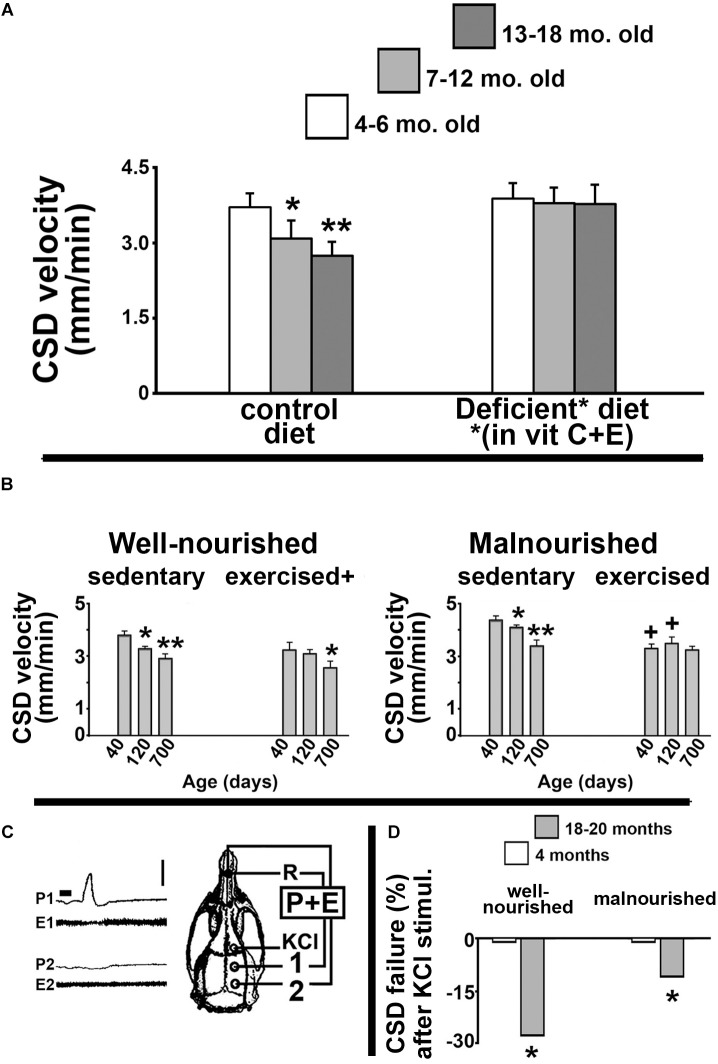
**(A)** Propagation velocity (in mm/min) of cortical spreading depression (CSD) measured in rats from three age groups: 4–6-month, 7–12-month, and 13–18-month-old animals. Data are the means ± standard deviations from eight animals per age group. The inverse correlation between CSD velocity and age (left panel) disappeared when animals were fed a diet that was deficient in the antioxidant vitamins C and E (right panel). ^∗^*p* < 0.05 compared to the 4–6-month-old group. ^∗∗^*p* < 0.005 compared to the two younger age-groups (ANOVA plus Holm-Sidak test). The skull diagram illustrates the area of KCl application to elicit CSD (in the frontal cortex), the two recording points (1 and 2, on the parietal cortex), and the point of placement of the common reference electrode (R, on the nasal bones). Data in this non-published panel **(A)** are from our previous publication (Guedes et al., 1996). **(B)** Mean ± standard deviation of the CSD Propagation velocity (in mm/min) in well-nourished (left panel) and malnourished (right panel) rats. In both nutritional groups, three age subgroups were studied: 40, 120, and 700 days of life. ^∗^*p* < 0.05 compared with the corresponding 40-day-old group. ^∗∗^*p* < 0.05 compared with the 40-day- and 120-day-old groups. ^+^*p* < 0.05 compared with the corresponding sedentary groups. **(C)** Recordings of DC slow potential change (P) and electrocorticogram (E) of CSD illustrating failure of CSD propagation in a 700-day-old rat. Note that after being elicited by KCl, CSD reached the recording point 1, but failed to reach point 2. **(D)** Percentage of KCl-elicited CSD episodes that failed to propagate to the remote recording point (2). ^∗^*p* < 0.05 compared to the corresponding younger (4-month-old) group. Data in the non-published panels **(B,D)** are from our previous publication (Batista-de-Oliveira et al., 2012). Traces shown in part C corresponds to panel **B** of Figure 3 from the paper by Batista-de-Oliveira et al. (2012). These traces are here reproduced with permission (No. 4532461257722) from the publisher.

Our group has dedicated considerable effort investigating how the age of an organism influences its brain’s ability to propagate CSD. In our pioneer study (Guedes et al., 1996), we documented the CSD propagation velocity in rats as a function of the animal’s age. We found that CSD velocity significantly decreased as the animal’s age increased. Figure 1A illustrates these data, demonstrating a significant reduction of CSD velocity in 7–12-month-old and 13–18-month-old Sprague-Dawley rats compared to younger (4–6-month-old) animals. This negative correlation was also found in Wistar rats and in another rodent species, the Mongolian gerbil (*Meriones unguiculatus*), suggesting that the age effect on CSD is neither a particular feature of one rat strain, nor a species-specific characteristic (Guedes et al., 1996). Furthermore, dietary deficiency of antioxidant vitamins enhances CSD velocity more in old than in young groups (Figure 1A, right panel), suggesting the involvement of age-related mechanisms based on redox imbalance in the aged brain.

These data, which indicated an inverse correlation between age and CSD susceptibility, have been confirmed by various reports (e.g., Farkas et al., 2011; Batista-de-Oliveira et al., 2012). In rats, an age-dependent diminished frequency of K^+^-induced SDs and an increased latency between subsequent SD events have also been reported (Farkas et al., 2011). In humans who suffered acute brain injury, spontaneous SD episodes are reportedly less frequent in old compared to younger patients (Fabricius et al., 2006). When considered together, these data support the hypothesis of lower SD susceptibility in the aged brain.

### Age, Ethanol Consumption, and CSD Features

Increased generation of reactive oxygen species (ROS) in the nervous tissue seems to modulate CSD susceptibility (Netto and Martins-Ferreira, 1989; El-Bachá et al., 1998; Mendes-da-Silva et al., 2014). A scenario of increased ROS production is certainly present in organisms under chronic ethanol consumption (Brocardo et al., 2017; Reddy et al., 2017). Using various paradigms of ethanol administration, we investigated the effects of ethanol on CSD propagation in the rat cortex. Initially, we demonstrated that daily ethanol gavage for 7 days or longer (18 days) significantly accelerated CSD (Guedes and Frade, 1993; Abadie-Guedes et al., 2008). In support of the hypothesis of ROS involvement in CSD ethanol effects, a carotenoid extract from shrimp, which is rich in the antioxidant astaxanthin, protected rat brain against the effects of ethanol on CSD (Bezerra et al., 2005). Furthermore, treatment with pure astaxanthin produced the same antagonistic effect of the shrimp carotenoid extract on the ethanol-induced action on CSD in a dose-dependent manner (Abadie-Guedes et al., 2008). In contrast to chronic ethanol treatment and in line with previous data from others (Sonn and Mayevsky, 2001), acute administration of ethanol (single gavage) decelerated CSD, and this effect was antagonized by astaxanthin (Abadie-Guedes et al., 2012). More recently, we demonstrated that the non-carotenoid antioxidant alpha-tocopherol also antagonizes the effect of ethanol on CSD, suggesting that this antagonistic action is not a particular property of carotenoids (Abadie-Guedes et al., 2016). In line with this suggestion, it is interesting to note that another non-carotenoid antioxidant molecule, ascorbic acid, has been shown to modulate CSD (Mendes-da-Silva et al., 2014, 2018).

Within the two age ranges that we investigated (60–80 and 150–180 days), no influence of age on the ethanol-induced action on CSD was observed (Abadie-Guedes et al., 2016). This is illustrated in Supplementary Figure S1. However, it remains to be investigated whether ethanol consumption at more advanced ages would affect CSD features. The hypothesis of an antioxidant-based modulation of CSD effects produced by ethanol shall be further investigated to understand their molecular mechanisms.

### Age, Physical Exercise, and CSD Features

In gerontology, neurological dysfunctions that are causally related to the aging process constitute a matter of increasing concern. Therapies that are based on physical exercise usually produce very positive outcomes (Marcelino et al., 2013; Fattoretti et al., 2018; Morgan et al., 2018). In a previous study (Batista-de-Oliveira et al., 2012), we investigated whether physical exercise and unfavorable lactation could modulate the age effects on brain electrophysiological signaling, as indexed by CSD. Data are summarized in Figure 1B. Compared with age-mated sedentary controls, exercised animals presented with significantly lower CSD propagation velocity (Figure 1B, left panel). Malnutrition induced by unfavorable lactation modulated the CSD effect of exercise (Figure 1B, right panel). Furthermore, the percentage of propagation failure of KCl-elicited CSD was significantly higher in older compared to younger animals (Figure 1C,D). Using the same unfavorable lactation paradigm, we documented a brain effect of exercise in increasing microglial (Iba1) immunolabeling in cortical tissue (Lima et al., 2014). We also demonstrated age-related reduced performance in both spatial and object identity recognition tasks, as well as changes in the innate immune system in the brain, with a significant impact on microglial homeostasis in aged rats (Viana et al., 2013).

### CSD-Induced Potentiation of Brain Signaling: Does Aging Play a Modulating Role?

In his pioneer seminal study, Leão noted that during CSD the spontaneously recorded electrocorticographic activity was substituted with abnormal, high-amplitude “epileptiform waves” (Leão, 1944a), suggesting a CSD-related imbalance in neuronal excitability. Five decades later, a post-CSD potentiation of cortical evoked responses was described *in vitro* (Footitt and Newberry, 1998). This was followed, a few years later, by demonstration of a similar CSD-dependent potentiation *in vivo*, both in non-mammal and mammal vertebrates, frog (Guedes et al., 2005) and rat (Souza et al., 2011, 2015, 2016; Silva-Gondim et al., 2017), respectively. Supplementary Figure S2 illustrates this potentiation effect in rats that were subjected to swimming exercise at two distinct ages: 8–23 and 60–75 days of life (Silva-Gondim et al., 2017). On average, after fully recovering from CSD, ECoG activity was shown to present a 14% to 43% higher amplitude than in the pre-CSD period for the same animal (*p* < 0.05). Early swimming and late swimming reduced and enhanced the post-CSD ECoG potentiation, respectively, compared to the respective non-exercised groups (*p* < 0.05), suggesting a differential effect of age of swimming exercise on CSD-induced ECoG potentiation.

## Final Remarks

### Cellular Mechanisms and Physiological Interpretation

In the aged brain, physiological and pathological conditions can modulate the electrophysiological mechanisms involved in brain signaling (Guedes et al., 1996; Farkas et al., 2011) and brain ability to produce and propagate CSD (Hertelendy et al., 2018). These last authors discussed the possibility that aging may impair physiologic processes such as ion channel function and ion pump activity that can be implicated in elicitation and propagation of CSD. Descriptions of the two-dimensional pattern of CSD propagation, based on magnetoencephalography (Eiselt et al., 2004) and optical imaging recording (Fujita et al., 2016), suggest an inhomogeneity of the cortical tissue, which also include age-related vascular hyporesponsivity and neuronal and glial density changes (Farkas et al., 2011; Hertelendy et al., 2018). These factors could be affected by aging and therefore influence the variability of CSD parameters. Furthermore, changes in the myelination pattern of the cortical tissue have been shown to inversely correlate with the propagation velocity of CSD, which might influence the stabilization and buffering of extracellular ion concentration that is crucial for CSD parameters (e.g., propagation velocity and DC amplitude) and cortical excitability, respectively (Merkler et al., 2009). Taken together, these pieces of evidence suggest that age-related alterations in the structural and functional complexity of the brain could play an important role in modifying CSD features in the aged brain.

### Application of the CSD Model to Investigate Factors That Disturb Normal Neuronal and Glial Functioning

As we have suggested in this review, experimental data allow us to conclude that CSD represents a useful and very important model to help understand how the brain modifies its functioning under physiological and pathological conditions, although CSD may not be the most appropriate read-out of the age-related adaptation of neuronal function. Because of ethical limitations, CSD studies on the normal human brain are not available so far. Occurrence of CSD in man has been usually reported in patients with severe neurological damage (Hartings et al., 2017; Lauritzen and Strong, 2017). From a translational perspective and within the limitation and caution that the extrapolation of experimental data to the human condition requires, our findings collectively support the hope that understanding CSD mechanisms could facilitate the development of more effective treatments against some aging-associated neurological disorders (Lauritzen et al., 2011). Furthermore, the CSD model might give us valuable insights into the molecular mechanisms through which, in the aged brain, factors such those here discussed (ethanol consumption, malnutrition and physical exercise) can modulate brain signaling. Another factor that shall be investigated in the aged brain using the CSD model is the reportedly beneficial effect of moderate consumption of caffeine-containing beverages, such as coffee, tea and guarana-based drinks (Vila-Luna et al., 2012; Perry et al., 2016; Mingori et al., 2017). Interestingly, recent findings suggest a facilitating effect of caffeine on CSD propagation in the young adult rat (Chagas et al., 2018). The possibility that caffeine ingestion actually represents a way of preventing or at least minimizing age-related brain dysfunction (Cunha and Agostinho, 2010) is an attractive theme that shall be explored in the near future. The relevance of this point can be viewed as very substantial when we consider, as highlighted in the introduction of this article, that the proportion of the human population that is affected by age-related neurological diseases is increasing over the last decades. Minimizing the negative consequences of brain aging on the quality of human life is a valuable goal that is worth being scientifically pursued.

## Author Contributions

RG and RA-G participated in all phases of conceiving, discussing, and writing this mini-review.

## Conflict of Interest Statement

The authors declare that the research was conducted in the absence of any commercial or financial relationships that could be construed as a potential conflict of interest.

## Supplementary Material

FIGURE S1 CSD features in ethanolc˛-treated Wistar rats of different ages. **(A)** recording of the slow DC potential change that is typical from CSD. Recordings are from two rats with 3 months (upper traces) and 6 months of life (lower traces). CSD was elicited by 1-min application (at the time marked with a black horizontal bar under trace 1) of a cotton ball (1-2 mm diameter) soaked in KCl 2% solution. One can note, in the older animal, the longer latency for a CSD wave to cross the interelectrode distance, indicating lower velocity, compared with the younger rat. **(B)** skull diagram showing the place of the reference electrode (R) on the nasal bones, the KCl application site (KCl) on the frontal bone, and the recording points 1 and 2 on the parietal bone. **(C)** velocity of CSD propagation in 3-months old (white bars) and 6-months old (gray bars) Wistar rats that were treated with water (control group) or 3 g/kg ethanol for 21 days. Data are expressed as mean ± standard deviation. ^∗^*p* < 0.05, ethanol group different from the water group. ^#^*p* < 0.05, 6-months old different from the 3-months old group. This unpublished figure is based on data from our previously published papers (Abadie-Guedes et al., 2012, 2016).

FIGURE S2 Examples of Electrocorticogram (ECoG) from three Wistar rats that are representative of the control group and two groups that were subjected to swimming exercise early in life (P8–P23), or late in life (P60–P75). ECoG after CSD presents higher amplitude compared with the ‘before CSD’ traces. This unpublished figure is based on data from our previously paper (Silva-Gondim et al., 2017).

TABLE S1 Examples of Brain morphological and physiological features that change as a function of aging.
