# Supplementary figures and images for: Brain Aging and Electrophysiological Signaling: Revisiting the Spreading Depression Model
Source: Front Aging Neurosci. 2019 Jun 7;11:136. doi: 10.3389/fnagi.2019.00136 (PMC6567796; doi:10.3389/fnagi.2019.00136)

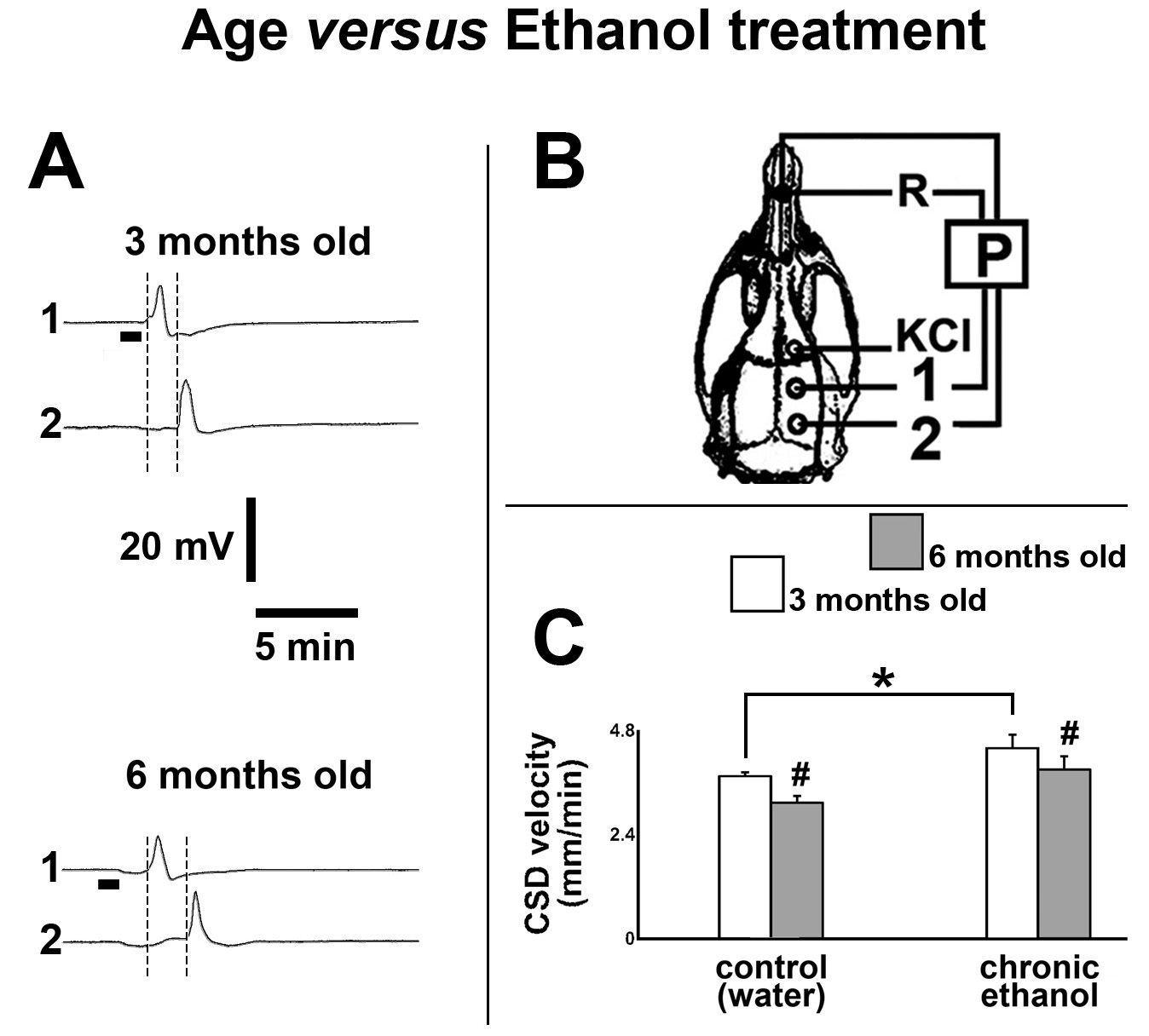

Supplement: FIGURE S1 — CSD features in ethanolc˛-treated Wistar rats of different ages. (A) recording of the slow DC potential change that is typical from CSD. Recordings are from two rats with 3 months (upper traces) and 6 months of life (lower traces). CSD was elicited by 1-min application (at the time marked with a black horizontal bar under trace 1) of a cotton ball (1-2 mm diameter) soaked in KCl 2% solution. One can note, in the older animal, the longer latency for a CSD wave to cross the interelectrode distance, indicating lower velocity, compared with the younger rat. (B) skull diagram showing the place of the reference electrode (R) on the nasal bones, the KCl application site (KCl) on the frontal bone, and the recording points 1 and 2 on the parietal bone. (C) velocity of CSD propagation in 3-months old (white bars) and 6-months old (gray bars) Wistar rats that were treated with water (control group) or 3 g/kg ethanol for 21 days. Data are expressed as mean ± standard deviation. ∗p < 0.05, ethanol group different from the water group. #p < 0.05, 6-months old different from the 3-months old group. This unpublished figure is based on data from our previously published papers (Abadie-Guedes et al., 2012, 2016). [file Image_1.TIFF]

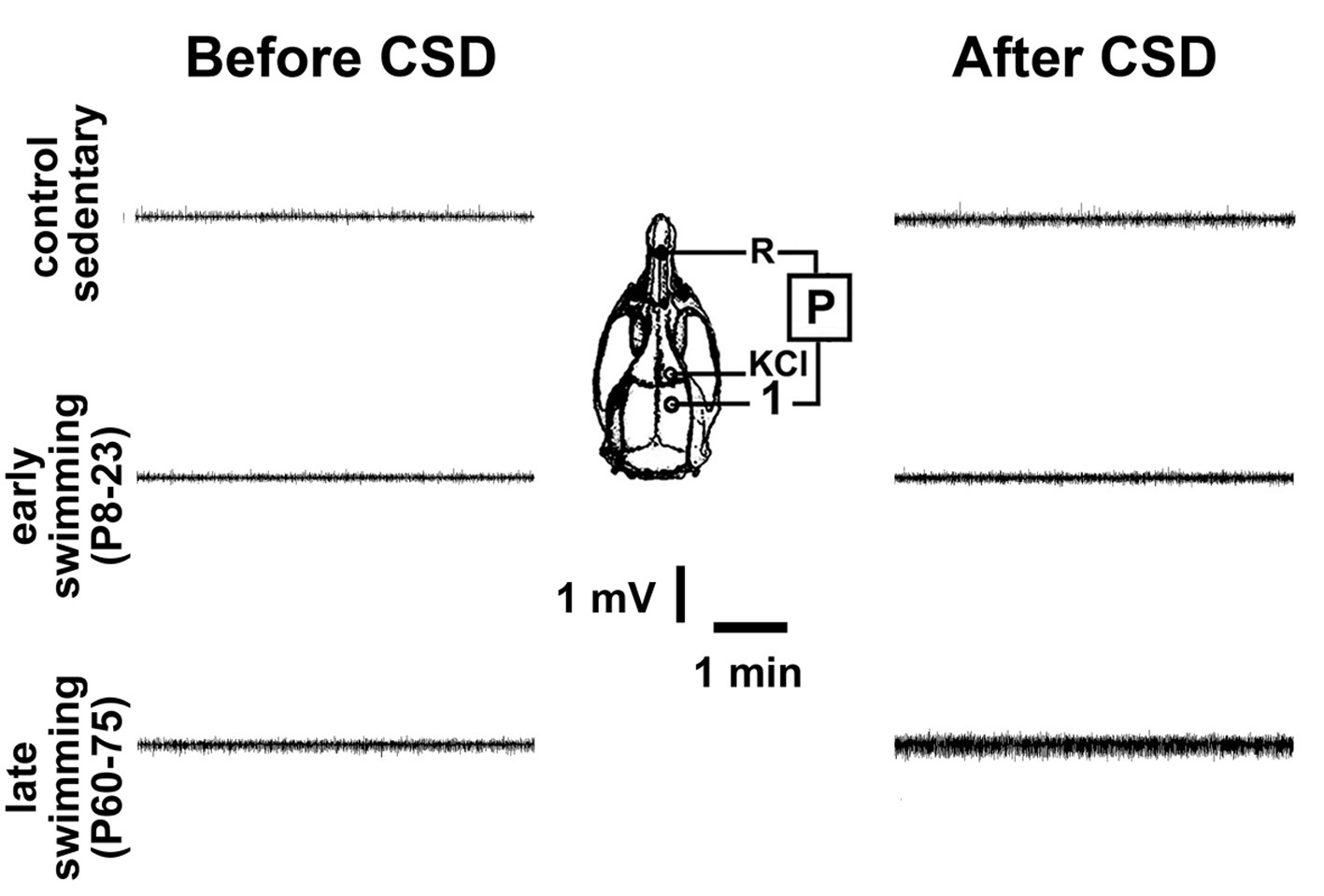

Supplement: FIGURE S2 — Examples of Electrocorticogram (ECoG) from three Wistar rats that are representative of the control group and two groups that were subjected to swimming exercise early in life (P8–P23), or late in life (P60–P75). ECoG after CSD presents higher amplitude compared with the ‘before CSD’ traces. This unpublished figure is based on data from our previously paper (Silva-Gondim et al., 2017). [file Image_2.TIFF]
