# Supplementary material for: Brain Aging and Electrophysiological Signaling: Revisiting the Spreading Depression Model
Source: Front Aging Neurosci. 2019 Jun 7;11:136. doi: 10.3389/fnagi.2019.00136 (PMC6567796; doi:10.3389/fnagi.2019.00136)
Supplement: TABLE S1 — Examples of Brain morphological and physiological features that change as a function of aging. [file Table_1.DOCX]

| **Examples of studies on the effects of aging on brain function** | | | |
| --- | --- | --- | --- |
| **Species** | **Reference** | **Condition** | **Main outcomes** |
| Human | Ferri et al., (2000) Clin. Neurophysiol. 111:591-599 | Somatosensory evoked potentials (SEP) were studied in subjects aged 7.1 to 17.2 years with typical centrotemporal spikes recorded during sleep; they were compared with age-matched normal controls. | Giant SEPs were not found in subjects older than 12 years; an age-related decrease in amplitude of giant SEPs as this age approached was observed. |
| Human | Musaeus et al. (2018) J. Alzh. Dis. 64:1359-1371 | Analysis of relative power of standard EEGs from 138 older health controls (OHC), 117 mild cognitive impaired (MCI), and 117 Alzheimer disease (AD) patients | Significant increases in the theta band and decreases in high frequency power in the temporal regions for eyes closed for AD and, for MCI compared to OHC |
| Human | Bourisly and Shuaib, (2018) Translat. Neurosci. 9:61-66 | Recording of event-related potential (ERP) in young and old healthy participants. | Age-related ERP (P200) with lower amplitude and longer latency than the young group. |
| Human | Zoubi et al. (2018) Front. Aging Neurosci. 10, no.184 | EEG data and machine learning (ML) methods were used in 468 patients to predict their age. | A reliable estimation of chronological age could be achieved with ML framework and extensive EEG signal features (correlation of 0.6 between the age and predicted age). |
| Human | Van der Zande et al. (2018) Front. Aging Neurosci. 10, no.190 | In 123 patients , EEGs were assessed visually, with Fast Fourier Transform (FFT), network and connectivity measures | EEG alteration was greater in patients with dementia with Lewi bodies (DLB) than in those with Alzheimer disease (AD). |
| Human | De Jong et al. (2018) Front. Aging Neurosci. 9, no. 1113 | In 24 young (18-30 y) and 24 midle-aged participants (50-67 y), measures based on typing behavior and EEG were recorded to assess subjective fatigue | Age influences the effects of mental fatigue on typewriting |
| Human | Catchlove et al. (2018) J. Exp. Neurosci. 12:1-11 | The authors assessed cerebrovascular reactivity (CVR) to CO2 inhalation in younger and aged adults, and the contribution of CVR to cognitive performance using blood-oxygen-level dependent contrast imaging (BOLD) fMRI. | When analyzing age groups separately, CVR in the hippocampus contributed significantly to memory score in the older group and was also related to subjective memory complaints. |
| Rhesus monkey | Ibañez-Contreras et al., (2018) Exp. Gerontol. 101:80-94 | In male and female monkeys from different ages, the authors evaluated Auditory, visual, and somatosensory evoked potentials, and correlated those variables with redox enzymatic activity from blood samples | Significant age-related differences in the distinct sensory pathways, and an increase in lipoperoxidation (TBARS) and the antioxidant enzymatic activities |
| Mouse | Baho & Di Cristo (2012) J. Neurosci.32:911-918. | The authors studied the synaptic development of GABAergic cortical interneurons using reconstruction methods, in cultured slices | A specific and age-dependent role of neural activity and neurotransmission levels in the establishment of the synaptic territory of GABAergic cells. |
| Mouse | Bell et al. (2010) Neuron 68:410-427 | In pericyte-deficient mice with different ages, the authors studied neurovascular functions | Pericyte loss leads to reduction in brain microcirculation, and blood-brain barrier breakdown |
| Rat | Buzsáki et al (1988) Neuroscience 26:735-744 | Electroencephalographic activity of the neocortex was evaluated in young (5–7 months) and aged (26–28 months) rats. | 1-Reduced power of high-frequency activity in the aged rats. 2-Scopolamine-induced power increase in all frequency bands, which in the higher-frequency range was less in the aged group. 3- In aged animals, higher incidence of high-voltage spindles, which lasted longer.. |
| Mouse | Etcheto et al., (2018) Mol Neurobiol 55: 7327 | Wild type and APPswe/PS1dE9 mice received either chow or High-Fat Diet (HFD) until 6 months of age, and they were treated with memantine (MEM)- (30 mg/kg) during the last 12 weeks. | MEM improves metabolic consequences of HFD. Treatment also improves animals learning abilities and decreases memory loss. |
| Monkey fibroblasts | Ritter et al., (2018) Front Neurosci 12:202. DOI: 10.3389/fncel.2018.00202 | The authors analyzed Tau Expression and cell death in vitro. | The proportion of mutant N279K Tau increases compared with wild-type at the cell nucleus |
